# Atrioventricular Left Ventricular Apical Pacing Improves Haemodynamic, Rotational, and Deformation Variables in Comparison to Pacing at the Lateral Wall in Intact Myocardium: Experimental Study

**DOI:** 10.1155/2014/316290

**Published:** 2014-02-09

**Authors:** Savvas Toumanidis, Anna Kaladaridou, Dimitrios Bramos, Elias Skaltsiotes, John Agrios, Constantinos Pamboucas, George Kottis, Anna Antoniou, Elektra Papadopoulou, Spyridon Moulopoulos

**Affiliations:** Department of Clinical Therapeutics, Medical School, National and Kapodistrian University of Athens, “Alexandra” Hospital, 80 Vas. Sofias Avenue, 11528 Athens, Greece

## Abstract

*Aim.* To assess the effect on left ventricular (LV) function of atrioventricular (AV) and ventricular pacing at the LV apical or lateral wall and to compare the normal torsional and deformation pattern of the intact LV myocardium with those created by the aforementioned LV pacing modes and sites. *Methods.* Experiments were conducted in pigs (*n* = 21) with normal LV function to investigate the acute hemodynamic effects of epicardial AV and ventricular LV pacing at the LV apical or lateral wall. Torsional and deformation indices of LV function were assessed using speckle tracking echocardiography. *Results.* AV pacing at the apex revealed a significant reduction in the radial strain of the base (*P* < 0.03), without affecting significantly the ejection fraction and the LV torsion or twist. In contrast, AV pacing at the lateral wall produced, in addition to the reduction of the radial strain of the base (*P* < 0.01), significant reduction of the circumferential and the radial strain of the apex (both *P* < 0.01) as well as of the ejection fraction (*P* < 0.002) and twist (*P* < 0.05). *Conclusions*. In pig hearts with intact myocardium, LV function is maintained at sinus rhythm level when AV pacing is performed at the LV apex.

## 1. Introduction

Temporary epicardial pacing is commonly indicated in cardiac surgical patients, when right ventricular (RV) and/or right atrial (RA) pacing wires are used to prevent postoperative bradyarrhythmia and atrioventricular (AV) block. Patients with atrial fibrillation receive only RV pacing wires, which conventionally have been used because of their easy application to the anterior wall of the RV, prior to disconnection from cardiopulmonary bypass. There is growing interest in seeking methods that use different pacing modes and/or pacing sites to maximize the benefits and minimize the harmful effects of artificial cardiac stimulation on LV function [1]. In these patients, frequent echocardiographic investigation is needed to follow up the LV function, even if patients remain asymptomatic.

Novel two-dimensional speckle tracking echocardiography (STE) allows detailed evaluation of LV mechanics, including LV mechanical dyssynchrony, LV strain, and LV torsion [2, 3]. This technique provides important additional information for the selection of the optimal pacing site. The role of STE in the assessment of the effects of RV apical pacing on LV function and the upgrade from RV to biventricular pacing have been evaluated in few studies [4, 5]. Data based on STE comparing the effects of different LV pacing modes (dual chamber versus single chamber pacing) and sites on the LV mechanics, LV strain, and LV torsion are still lacking.

The aim of this study was to assess the effect on LV function of AV and ventricular LV pacing at the LV apical or lateral wall and to compare the normal torsional pattern of the intact LV myocardium with those created by the aforementioned LV pacing modes and sites.

## 2. Materials and Methods

The protocol complied with the “Principles for the Care of Experimental Animals” and the “Guidelines for the Care and Use of Experimental Animals” issued by the US National Academy of Sciences and National Institute of Health (version 85-23, revision 1996) and was approved by the Scientific Committee of the “Alexandra” University Hospital.

### 2.1. Surgical Preparation

Twenty-one healthy pigs, weighing 35 ± 5 kg, were sedated with intramuscular administration of midazolam 5 mg/kg and ketamine potassium 5 mg/kg, anesthetized with intravenous (IV) thiopental sodium 5 mg/kg, intubated, and controlled by mechanical ventilation (Sulla 808V, Drager Medizintechnik GmbH, Germany). Anesthesia was maintained with IV propofol 0.1-0.2 mg/kg. During the experiment, analgesia was maintained with the administration of opioid-fentanyl. Additional anesthetic was administered during the experiment as needed. A 7F sheath was inserted into the right internal jugular vein for the delivery of drugs and fluids. Fluid loss was compensated for by continuous infusion of saline into the right jugular vein. Through a left external carotid artery a 6F pigtail catheter was placed into the LV cavity and used for LV pressure monitoring. Lead II of the standard electrocardiogram (ECG), LV pressure, and hemoglobin oxygen saturation were monitored throughout the experiment. After catheter insertion, a 5000 IU heparin bolus was administered to avoid endovascular thrombus formation. Loading conditions were kept constant during the different maneuvers. A regular median sternotomy was performed after thymic resection and a longitudinal pericardiotomy was performed. Temporary myocardial pacing leads (Medtronic, type 6500, Minneapolis, Minnesota) were attached to the surface of the right atrium and to the epicardium of the LV apex and of the LV lateral wall (approximately 2 cm below the base). The leads were connected to an external pacemaker (Medtronic AV pacing System Analyzer Model 5311B). The two LV electrodes were connected to a two-channel external pulse stimulator (Medtronic model 2883), allowing setting of thresholds for each electrode separately and pacing through each of the electrodes separately. Pacing was unipolar with an indifferent electrode positioned in between the intercostal muscles. During AV pacing the AV delays were short enough to produce an activation wave originating from the ventricular pacing lead. Under all conditions, pacing was performed at about twice the stimulation threshold. The pacing rate was set at 10 beats/min above the intrinsic heart rate in each case.

### 2.2. Standard Echocardiography

The echocardiographic study was performed using a Vivid *i* digital ultrasound system (GE Medical Systems Ultrasound Israel Ltd., Tirat Hacarmel, Israel) and a 3.5 MHz phased array transducer. Two-dimensional gray-scale echocardiographic images were obtained using 2nd harmonic imaging. Instrument settings were held constant for each experiment. At each stage of the experiment, with or without pacing, the following parameters were measured [6]: LV end-diastolic and end-systolic long-axis (Ld, Ls) and short-axis (Sd, Ss) dimensions, the LV end-diastolic (EDV) and end-systolic (ESV) volumes, and the EF (modified Simpson's rule). Stroke volume (SV) and cardiac output (CO) were measured and calculated from a subxiphoid epicardial apical four-chamber view. Mitral early (*e*) diastolic flow velocity was measured and the early diastolic (*e*′) wave of the lateral mitral annulus was obtained by tissue Doppler imaging from the apical 4-chamber view. The ratio *e*/*e*′ was calculated. To determine the timing of cardiac events, mitral inflow and LV outflow were recorded using pulsed Doppler echocardiography. Three consecutive cardiac cycles were stored in cineloop format for offline analysis. Averaged values were calculated for each parameter.

### 2.3. Two-Dimensional Speckle Tracking Echocardiography

Assessment of LV rotation and twist was obtained by acquisition of specific short-axis planes with internal landmarks: the basal plane was acquired at the level of the mitral valve leaflets, while excluding the mitral annulus, and the apical plane was acquired distally to the papillary muscles. The frame rate range was 65–80/s. In each phase, three consecutive cardiac cycles' cineloop images were stored for offline analysis with a dedicated platform EchoPac PC (version 7.0, GE Medical Systems). The software automatically defined the ventricular centroid for the midmyocardial line on a frame-by-frame basis and calculated the time domain LV strain (radial and circumferential), rotation, and rotational velocity for each segment in both short-axis planes. Regional strain curves were then analyzed, and peak radial strain and peak circumferential strain were measured for each segment at both planes. The averaged LV rotation and rotational velocity profile were used for the calculation of LV twist and twist velocity. As viewed from the apex, counterclockwise rotation was expressed as a positive value, whereas a clockwise rotation was denoted as a negative value [7]. LV twist was defined as the net difference between apical and basal rotation in degrees (°). Because the degree of rotation for the same amount of LV torque increases as the distance from the midventricular level increases, LV twist is expected to vary with the distance between the planes at which basal and apical short-axis images are obtained. LV torsion (°/mm) was calculated as LV twist/Ld longitudinal length (measured between the locations of the base and apex of the LV in the end-diastolic phase) [8]. The opposite rotation after LV twist was defined as LV untwist and the time derivative of LV untwist was designated as the LV untwisting rate (°/s). The following parameters were measured: (1) peak apical and basal rotations and rates; (2) peak LV twist, torsion, torsion rate, and peak LV untwisting rate; and (3) peak apical and basal systolic radial and circumferential strains.

### 2.4. Experimental Protocol

Throughout the experiment, surface ECG lead II from the limb lead electrodes was monitored and recorded at a speed of 100 mm/s by a multichannel device (Dynamap Plus Vital Signs Monitor, Criticon, Tampa, FL, USA). A pigtail catheter was placed through an arterial sheath and advanced retrogradely across the aortic valve into the LV cavity for the measurement of LV pressure and peak rate of LV pressure increase (dP/dt_max⁡_). After completion of the surgical preparations, a steady-state period of 15 min was allowed. Baseline measurements were obtained in sinus rhythm and then repeated during ventricular pacing at the LV apex and the LV lateral wall and during dual AV pacing at the LV apex and the LV lateral wall, in random order. Measurements were performed after two minutes' pacing at each site separated by two-minute intervals in sinus rhythm.

Ten animals were randomly selected to assess the reproducibility of apical circumferential strain and Bland-Altman analysis was performed to evaluate intraobserver and interobserver agreement. The mean difference ± 2SD for apical circumferential strain was 0.4 ± 2.2% for intraobserver and 2.5 ± 3.6% for interobserver agreement.

### 2.5. Statistical Analysis

Results are presented as mean ± SD. Statistical analysis was performed on absolute values, and each experiment served as its own control. The normality of distributions was checked using the Kolmogorov-Smirnov test. One-way analysis of variance for repeated measurements was used to evaluate the significance of the effect of pacing mode and site on each variable. The Bonferroni correction was used for post hoc comparisons. Pearson correlations were studied and linear regression analysis was performed to define the relationship between parameters. The level of significance was set at *P* < 0.05. The statistical software package SPSS for Windows, version 20, was used for the analysis (SPSS Inc., Chicago, IL, USA).

## 3. Results

**Figure 1:**
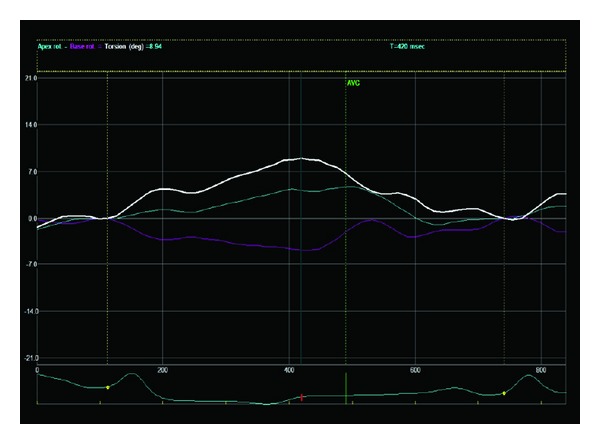
A representative case of left ventricular (LV) twist (8.94°) in sinus rhythm. The white color tracing denotes LV twist; blue denotes apical rotation (4.75°); purple denotes basal rotation. AVC: aortic valve closure.

**Figure 2:**
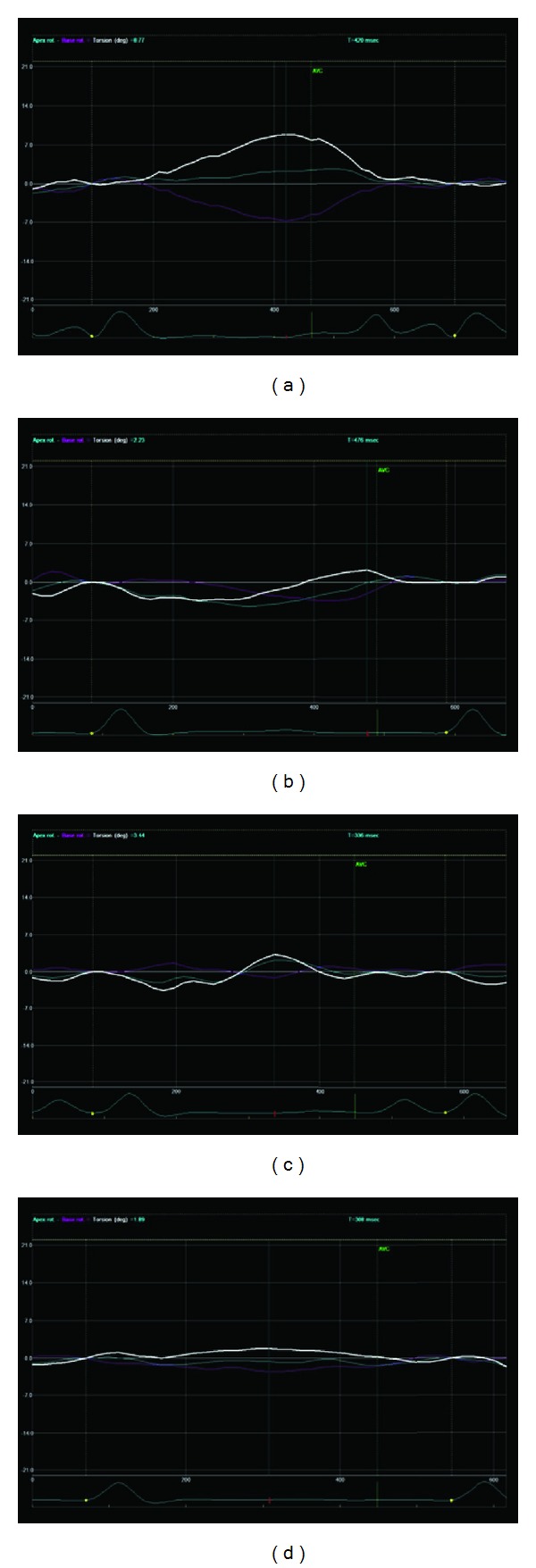
The same animal during atrioventricular pacing at the left ventricular (LV) apex (a), ventricular pacing at the LV apex (b), atrioventricular pacing at the lateral wall (c), and ventricular pacing at the lateral wall (d). LV twist was maintained at the same level as in sinus rhythm (Figure 1) when atrioventricular pacing was implemented at the LV apex. Atrioventricular or ventricular pacing at the lateral wall and ventricular pacing at the LV apex reduce twist significantly.

**Figure 3:**
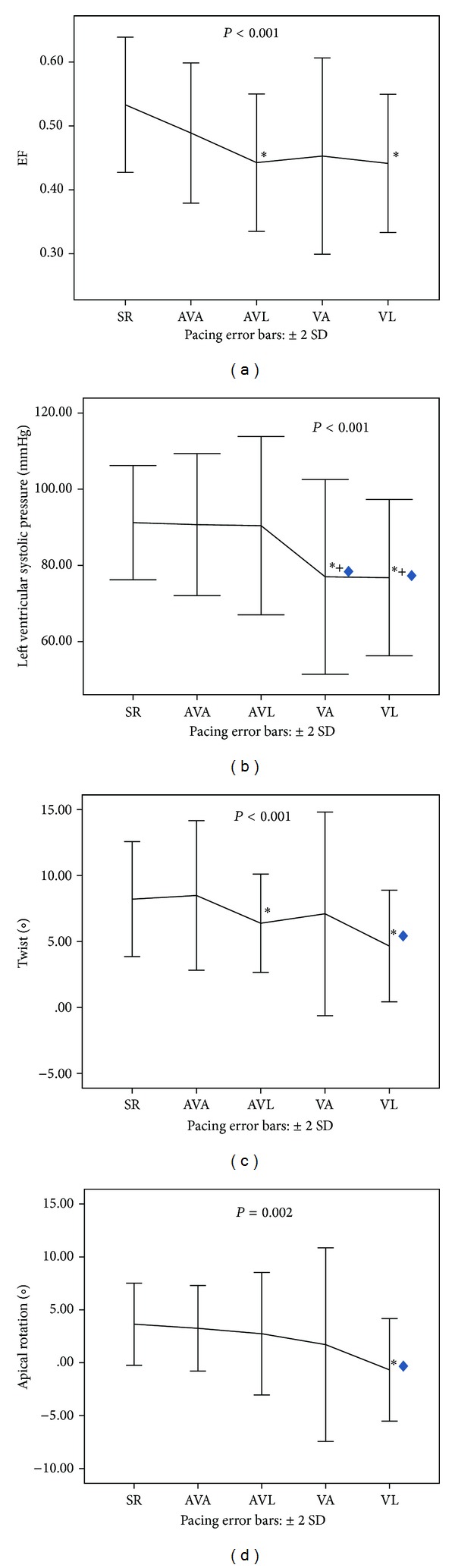
Diagrammatic pattern of echocardiographic ejection fraction (a), left ventricular (LV) systolic pressure measurement (b), twist (c), and apical rotation (d) obtained by speckle tracking imaging during sinus rhythm (SR), atrioventricular LV pacing at the apex (AVA) and lateral wall (AVL), and ventricular pacing at the apex (VA) and lateral wall (VL). Asterisk (∗) denotes that the mean difference from SR is significant at the 0.05 level; rhombus (*◊*) denotes significant differences from AVA and plus sign (+) from AVL.

Epicardial pacing of intact myocardium revealed impairment for LV function in comparison to sinus rhythm. Specifically, there were significant decreases in the LV indices of systolic function, SV, EF, LV systolic pressure (all *P* < 0.001), dP/dt_max⁡_ (*P* = 0.005), and the eccentricity index Ld/Sd (*P* = 0.02); in contrast, ESV (*P* < 0.05) and the diastolic function index *e*/*e*′ ratio, which denotes an elevated LV filling pressure (*P* = 0.04), increased significantly; dP/dt_min⁡_, which denotes ventricular lucitropy, decreased significantly (*P* < 0.001), (Table 1). Moreover, the torsional parameters (Figures 1 and 2), twist (*P* < 0.001) and torsion (*P* = 0.002), and apical rotation (*P* = 0.002) and apical peak systolic rotation rate (*P* = 0.005), as well as the deformation variables, radial strain of the apex (*P* < 0.01) and the base (*P* = 0.004), together with circumferential strain of the apex (*P* < 0.001) and the base (*P* = 0.02), deteriorated significantly (Table 2, Figure 3).

### 3.1. Pacing Site

AV pacing at the apex showed the least significant changes for LV function in comparison to sinus rhythm (Tables 1 and 2). The main changes concerned primarily a significant reduction in the radial strain of the base (*P* = 0.03), without affecting significantly the EF and the LV torsion or twist. In contrast, AV pacing at the lateral wall produced, in addition to the reduction of the radial strain of the base (*P* < 0.01), significant reduction of the circumferential and the radial strain of the apex (both *P* < 0.01) as well as of the EF (*P* = 0.002), torsion (*P* = 0.04), and twist (*P* < 0.05).

Ventricular pacing at the apex showed less significant changes than pacing at the lateral wall in comparison to sinus rhythm. It produced a significant reduction of the SV and LV systolic pressure (both *P* < 0.001) and peak systolic circumferential strain of the apex (*P* = 0.04), while pacing at the lateral wall also led to a reduction in SV and LV systolic pressure (both *P* < 0.001), while producing a significant decrease in EF (*P* < 0.001), dP/dt_max⁡_ (*P* < 0.01), dP/dt_min⁡_ (*P* < 0.001), apical rotation (*P* < 0.001) and rotation rate (*P* = 0.03), and LV torsion (*P* = 0.007) or twist (*P* = 0.003).

### 3.2. Pacing Mode

AV pacing showed only minor changes in comparison to sinus rhythm (Tables 1 and 2). AV pacing at the apex produced a significant reduction only in the radial strain of the LV base, while ventricular pacing at the same site significantly reduced the SV, LV systolic pressure, and the circumferential strain of the apex. Moreover, AV pacing at the lateral wall showed a significant reduction in EF and the circumferential and the radial strain of the apex, as well as the radial strain of the base and the LV twist or torsion. Ventricular pacing at the same site produced a significant reduction in EF, SV, dP/dt_max⁡_, dP/dt_min⁡_, LV systolic pressure, apical rotation and its rotation rate, and LV torsion or twist. Ventricular pacing at the lateral wall was revealed to be inferior not only to sinus rhythm but also to LV AV pacing at the apex, since LV systolic pressure (*P* < 0.001), torsion, or twist (both *P* = 0.04), as well as rotation and rotation rate of the apex (both *P* = 0.02) were all significantly lower.

For every pacing mode and pacing site the heart rate was significantly higher and the QRS duration significantly longer in comparison to sinus rhythm (*P* < 0.001 all).

**Figure 4:**
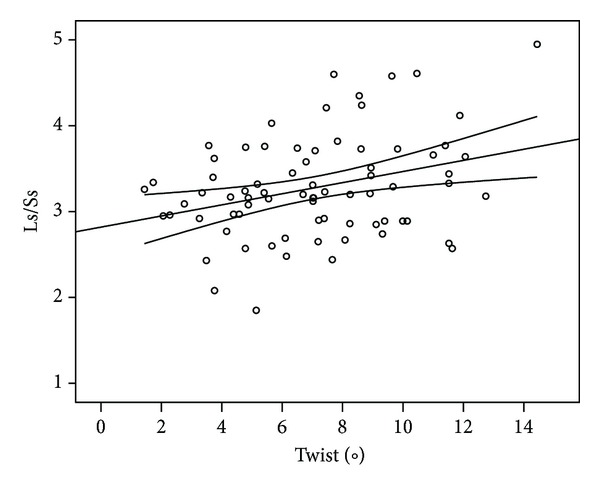
Diagram showing the positive correlation (*r* = 0.32, *P* = 0.005) between LV systolic twist and the index of acute LV remodeling systolic Ls/Ss ratio during temporary LV pacing for every pacing mode and site (LV: left ventricle, Ls/Ss: LV end-systolic long- (Ls) and short-axis (Ss) dimensions ratio).

EF was correlated negatively with pacing and QRS duration and positively with peak systolic radial strain of the apex (Table 3) while dP/dt_max⁡_ was affected negatively by heart rate and positively by LV systolic pressure, apical rotation, and the eccentricity index Ls/Ss. Of the torsional parameters, twist was found to correlate positively with Ls/Ss ratio (Figure 4), twisting and untwisting rate, the clockwise rotation and rotation rate of the base, and the counterclockwise rotation and rotation rate of the apex; twist was negatively correlated with pacing, QRS duration, heart rate, and the *e*/*e*′ ratio. In particular, apical rotation was correlated positively (in addition to the twist relationships) with LV systolic pressure and dP/dt_max⁡_.

Multivariate regression analysis showed that EF was independently related to pacing (*b* = −0.07, *P* < 0.001) while twist was independently affected by apical rotation rate (*b* = 0.04, *P* < 0.001), untwisting rate (*b* = −0.03, *P* < 0.001), and heart rate (*b* = −0.05, *P* < 0.001). Rotation of the apex and base was excluded from the analysis.

## 4. Discussion

The present study, performed in pig hearts with normal ventricular conduction, demonstrates that LV function was maintained at the same level as in sinus rhythm when AV pacing was implemented at the LV apex. AV or ventricular pacing at the lateral wall and ventricular pacing at the LV apex reduce LV function significantly. The hemodynamic superiority of LV apical AV pacing in comparison to AV pacing at the lateral wall may be explained by a relatively physiological sequence of electrical activation when pacing from these sites, while ventricular pacing at the apex or lateral wall suffers from the lack of the “atrial kick.”

In view of the potential detrimental effects of RV apical pacing on LV function, different approaches to the selection of pacing mode and site have been explored to prevent or diminish these effects [1, 9]. Experimental [10, 11] and human studies [12] have suggested that single-site LV pacing improved LV function compared with RV apical pacing. Furthermore, the impact of LV pacing on LV function is also affected by the site of stimulation [9, 13]. A few recent studies suggest that LV apical pacing can improve cardiac performance similar to biventricular pacing [11, 14, 15]. Vichova et al. showed that AV pacing at the LV apex significantly reduced LV dyssynchrony without a significant increase in cardiac output in comparison with RA-RV pacing [16]. To date, there is no clear consensus as to the optimal strategy regarding LV lead placement. In this setting, two-dimensional STE strain imaging may provide important additional information for the selection of the optimal pacing mode and site. Unfortunately, no data from direct comparisons of the effects of different LV pacing modes and sites on LV strain and LV torsion are yet available.

The results of the present study, comparing the impact of various temporary LV pacing modes and sites on indices of LV systolic and diastolic function using hemodynamic, strain, and torsional analysis, demonstrate that LV pacing mode and lead location have a significant impact on LV function. In particular, LV pacing produced a significant deterioration in the main hemodynamic (EF, dP/dt_max⁡_, dP/dt_min⁡_, ESV, SV, Ld/Sd ratio, LVSP, and *e*/*e*′ ratio), torsional (twist or torsion, apical rotation and rotation rate), and deformation (radial and circumferential strain of the LV base and apex) indices of LV function. During sinus rhythm, the electrical impulse travels from the rapid His-bundle conduction system towards the apex. During LV pacing, activation is more asynchronous due to the longer QRS duration. This widening of the QRS duration is primarily due to the propagation of the electrical impulse through the slowly conducting myocardium instead of the His-Purkinje system. QRS widening negatively affects EF, twist, and apical rotation. AV pacing at the LV apex provides a fairly physiological sequence of stimulation, although activation is more asynchronous due to longer QRS duration. However, a good sequence of electrical activation is sufficient to allow a near normal LVEF for AV and ventricular apical pacing but not for pacing at the lateral wall, although QRS duration did not differ significantly between them. The importance of a proper sequence of activation is further supported by the finding that, in normal hearts, biventricular and multisite pacing does not improve LV function as compared with LV apex pacing alone, even though QRS duration is shorter in the former pacing modes [11]. Some studies reported a correlation between QRS duration and systolic LV function, but such correlation was absent in other studies. The positive correlation between EF and apical radial strain indicates that radial strain is an accurate index of LV systolic function demonstrated by two-dimensional STE. The LV dP/dt_max⁡_ changes produced by pacing were correlated with the acute remodeling of the LV, as expressed by the changes in Ls/Ss ratio, while a more spherical LV is associated with depressed systolic function. Moreover, a reduced apical rotation during pacing was correlated with a reduced dP/dt_max⁡_. The data from the present study extend earlier findings on the beneficial hemodynamic effects of LV apical pacing in an acute hemodynamic study [11]. Moreover, our findings on LV apical pacing are in line with the superior hemodynamic performance found in an acute pediatric pacing study [14]. Its chronic benefit was demonstrated by the reversal of heart failure after switching from RV pacing to LV apical pacing in a 2-year-old patient [17]. In these pediatric studies, the contraction sequence and LV pump function during single-site LV apical pacing were at least as good as during biventricular pacing. The favorable hemodynamic effect of LV apical pacing has been recognized before [18, 19], but the findings with regard to LV torsional and strain parameters are new.

### 4.1. Effect of LV Pacing on Torsional and Deformation Indices

Two-dimensional STE, validated against sonomicrometry and tagged-magnetic resonance imaging, can provide reliable and accurate information [2, 3, 20]. Nonetheless, studies based on STE that compare the effects of different LV pacing modes and sites on LV mechanics are lacking. A previous study that examined the effect of RV apical pacing on LV torsion with MRI tagging showed that peak basal and peak apical rotation decreased significantly. As a result, LV torsion decreased significantly. Notably, this decrease in LV torsion may result in less efficient LV filling and emptying and contribute to LV systolic and diastolic dysfunction during RV apical pacing [21]. Data on the effects of different LV pacing sites on LV torsion are limited. Changes in torsion during abnormal electrical activation of the LV by LV pacing can be expected.

The present study showed that LV pacing had a detrimental effect on LV torsion or twist. However, the physiologic propagation of the electrical impulse from the apex during AV or even apical ventricular pacing did not change LV torsion or twist significantly. In contrast, torsion and twist during ventricular pacing at the lateral wall were significantly lower, compared not only to sinus rhythm but also to AV pacing at the apex. This effect seems to be mainly due to the adverse effects on the apical rotation and peak systolic rotation rate and not to the rotation of the base, which was not affected significantly. In particular, ventricular pacing at the lateral wall produced mild clockwise rotation of the apex. The negative effect of pacing on the LV twisting capability and apical rotation is accompanied by the known factors of heart rate, eccentricity index Ls/Ss ratio and the index of LV filling pressure, and the *e*/*e*′ ratio.

In addition to changes in hemodynamic and torsional parameters, the absolute strain values may be affected by LV pacing mode and site [5]. At present no studies are available that have assessed the effects of LV AV and ventricular pacing at the apical or lateral wall on radial and circumferential apical and basal strain with STE. LV pacing significantly reduced both apical and basal radial and circumferential strain. More studies are needed to fully understand the effect of LV pacing on LV radial and circumferential strain as assessed with STE.

### 4.2. Limitations

The finding that the optimal pacing site was more different in normal than in pig hearts with acute myocardial infarction [13] indicates that extrapolation of the data from the present study in intact pig hearts to diseased hearts should be done with care.

### 4.3. Clinical Implications

The results of our study may have important clinical implications. Temporary LV pacing of an intact myocardium, a common situation in cardiac surgical patients, is “necessary evil.” However, improved hemodynamic function during pacing in bradycardia-pacing patients is desirable. Maintaining LV pump function as close as possible to sinus rhythm, as demonstrated for LV AV apical pacing, may decrease the long-term risk of developing heart failure.

The present study shows that it is possible to achieve good cardiac function by pacing from a single site, such as the LV apex. This pacing modality may be interesting, since it requires fewer epicardial leads than biventricular pacing. The feasibility of the use of these novel alternate pacing sites clearly depends on the availability of tools for easy and safe implantation. LV apical pacing does require an open thorax or a minimally invasive approach. Consequently, LV apical pacing currently is limited to children (where pacing leads are often placed epicardially) and patients undergoing surgical implantation. However, the LV apex is relatively easy to access using a subxiphoid approach, so that, after the development of proper tools, positioning of a pacing lead at this site could become feasible for many more patients. Moreover, with the currently available pacing leads, the LV apex could be reached using the transcoronary venous approach if the lead can be advanced far enough. AV pacing at the LV apex may help to reduce the morbidity and mortality after cardiac surgery. Its early postoperative use may reduce the need for postoperative inotrope support.

## 5. Conclusions

In pig hearts with intact myocardium, LV function is maintained at sinus rhythm level when AV pacing is performed at the LV apex, possibly because pacing from this site creates a more physiological propagation of electrical conduction. Further studies are necessary to prove the beneficial effects of AV pacing at the LV apex on the postoperative morbidity and mortality after cardiac surgery.

## Figures and Tables

**Table 1:** Comparison of the hemodynamic and conventional echocardiographic variables between sinus rhythm and pacing in various modes and sites.

	SR	AVA	AVL	VA	VL	*P*
Ld/Sd ratio	2.56 ± 0.38	2.35 ± 0.28	2.40 ± 0.25	2.33 ± 0.26	2.37 ± 0.34	0.02
Ls/Ss ratio	3.34 ± 0.55	3.23 ± 0.61	3.13 ± 0.46	3.12 ± 0.56	3.03 ± 0.51	0.18
EDV (mL)	62.25 ± 10.76	56.73 ± 13.96	62.81 ± 14.41	56.39 ± 11.33	56.03 ± 11.44	0.11
ESV (mL)	29.16 ± 6.52	28.80 ± 6.63	34.78 ± 7.28	31.17 ± 8.00	31.55 ± 7.63	0.05
SV (mL)	33.09 ± 5.95	27.93 ± 8.50	28.03 ± 8.70	25.23 ± 5.58*	24.49 ± 5.19*	0.001
EF (%)	0.53 ± 0.05	0.49 ± 0.05	0.44 ± 0.05*	0.45 ± 0.08	0.44 ± 0.05*	0.001
CO (mL/min)	3064 ± 819	3001 ± 1267	2986 ± 1149	2777 ± 940	2719 ± 835	0.45
SP (mmHg)	91 ± 7	90 ± 9	90 ± 11	77 ± 12^∗*♦*+^	76 ± 10^∗*♦*+^	0.001
dP/dt_max _(mmHg/s)	1.41 ± 0.23	1.31 ± 0.36	1.35 ± 0.27	1.16 ± 0.30	1.18 ± 0.21*	0.005
dP/dt_min_ (mmHg/s)	−1.54 ± 0.35	−1.42 ± 0.52	−1.40 ± 0.35	−1.33 ± 0.49	−1.15 ± 0.36^∗+^	0.001
HR (beats/min)	92 ± 18	106 ± 13*	105 ± 16*	110 ± 17*	111 ± 15*	0.001
QRS (ms)	71 ± 5	106 ± 20*	102 ± 14*	108 ± 20*	108 ± 16*	0.001
*e*/*e*′ ratio	8.59 ± 3.24	7.90 ± 2.77	11.18 ± 3.12	9.78 ± 5.29	11.74 ± 4.11	0.04

Values are mean ± SD. *P* indicates the overall significance of the effect of pacing mode and site on each variable by one-way analysis of variance for repeated measurements. Symbols (*, ^♦^, ^+^) relate to individual group comparisons.

*Asterisk denotes that the mean difference from sinus rhythm is significant at the 0.05 level.

^♦^Rhombus denotes that the mean difference is significant at the 0.05 level from atrioventricular pacing at the left ventricular (LV) apex.

^+^Plus sign denotes that the mean difference is significant at the 0.05 level from atrioventricular pacing at the LV lateral wall.

AVA: atrioventricular pacing at the LV apex; AVL: atrioventricular pacing at the LV lateral wall; CO: cardiac output; EDV: LV end-diastolic volume; EF: LV-ejection fraction; ESV: LV end-systolic volume; *e*: mitral early filling velocity; *e*′: early diastolic mitral annulus velocity by tissue Doppler imaging; HR: heart rate; Ld: LV end-diastolic long-axis dimension; Ls: LV end-systolic long-axis dimension; SP: LV systolic pressure; Sd: LV end-diastolic short-axis dimension; SR: sinus rhythm; Ss: LV end-systolic short-axis dimension; SV: LV-stroke volume; VA: ventricular pacing at the LV apex; VL: ventricular pacing at the lateral wall.

**Table 2:** Comparison between left ventricular torsional and strain parameters during sinus rhythm and in relation to various pacing modes and sites.

	SR	AVA	AVL	VA	VL	*P*
Torsion (°/mm)	0.13 ± 0.04	0.13 ± 0.05	0.10 ± 0.03*	0.11 ± 0.06	0.07 ± 0.03^∗*♦*^	0.002
Twist (°)	8.21 ± 2.18	8.49 ± 2.83	6.38 ± 1.86*	7.10 ± 3.86	4.66 ± 2.12^∗*♦*^	0.001
Torsion rate (°/s)	53.79 ± 19.21	71.59 ± 24.25	60.35 ± 22.65	67.52 ± 22.36	57.37 ± 31.56	0.27
Untwisting rate (°/s)	−79.54 ± 27.73	−94.83 ± 35.22	−81.01 ± 46.52	−74.66 ± 27.39	−62.97 ± 28.86	0.16
LV base						
Rotation (°)	−5.33 ± 1.67	−4.93 ± 2.32	−3.17 ± 3.14	−5.28 ± 1.60	−4.96 ± 1.77	0.09
Rotation rate (°/s)	−39.28 ± 18.20	−50.98 ± 16.14	−37.30 ± 28.42	−52.33 ± 17.10	−56.87 ± 27.03	0.14
Radial strain	44.99 ± 13.88	31.90 ± 15.74*	27.90 ± 9.92*	32.36 ± 9.58	34.95 ± 14.18	0.004
Circum. strain	−11.54 ± 4.22	−10.34 ± 2.44	−10.49 ± 2.69	−11.74 ± 3.38	−13.03 ± 30^*♦*^	0.02
LV Apex						
Rotation (°)	3.64 ± 1.94	3.25 ± 2.02	2.74 ± 2.89	1.71 ± 4.57	−0.68 ± 2.43^∗*♦*^	0.002
Rotation rate (°/s)	26.61 ± 10.12	36.30 ± 27.23	26.28 ± 24.40	15.93 ± 46.09	−6.65 ± 30.15^∗*♦*^	0.005
Radial strain	39.35 ± 13.80	30.66 ± 9.55	24.78 ± 6.88*	27.31 ± 11.02	30.80 ± 10.72	0.01
Circum. strain	−16.74 ± 3.34	−12.74 ± 3.93	−11.71 ± 3.31*	−13.91 ± 3.09*	−13.66 ± 3.65	0.001

Values are mean ± SD. *P* indicates the overall significance of the effect of pacing mode and site on each variable by one-way analysis of variance for repeated measurements. Symbols (*,^♦^) relate to individual group comparisons.

Circum.: circumferential. Other abbreviations and symbols as in Table 1.

**Table 3:** Significant correlations between hemodynamic, torsional, and deformation indices in the overall study population.

Variables	EF (%)		dP/dt_max_		Twist (°)		Apical rotation (°)	
	*r*	*P*	*r*	*P*	*r*	*P*	*r*	*P*
Pacing	−0.46	0.001			−0.27	0.02	−0.29	0.01
Ls/Ss ratio			0.23	0.04	0.32	0.005	0.34	0.003
QRS duration (ms)	−0.29	0.01			−0.26	0.02	−0.36	0.002
HR (beats/min)			−0.30	0.01	−0.28	0.02	−0.23	0.04
*e*/*e*′ ratio					−0.23	0.05		
LVSP (mmHg)			0.65	0.001			0.36	0.002
dp/dt_max _(mmHg/s)							0.26	0.03
Twist (°)							0.71	0.001
Twisting rate (°/s)					0.53	0.001	0.29	0.01
Untwisting rate (°/s)					−0.64	0.001	−0.34	0.003
Apical rotation (°)			0.26	0.03	0.71	0.001		
Apical rotation rate (°/s)					0.55	0.001		
Basal rotation (°)					−0.58	0.001		
Basal rotation rate (°/s)					−0.37	0.001		
Basal radial strain					0.28	0.02	0.24	0.04
Apical radial strain	0.26	0.02						

Abbreviations as in Tables 1 and 2.
